# Gaps in Border Controls Are Related to Quarantine Alien Insect Invasions in Europe

**DOI:** 10.1371/journal.pone.0047689

**Published:** 2012-10-24

**Authors:** Steven James Bacon, Sven Bacher, Alexandre Aebi

**Affiliations:** 1 Biodiversity and Environmental Management, Agroscope Reckenholz-Tänikon ART, Zürich, Switzerland; 2 Ecologie et Evolution, Université de Fribourg, Fribourg, Switzerland; La Trobe University, Australia

## Abstract

Alien insects are increasingly being dispersed around the world through international trade, causing a multitude of negative environmental impacts and billions of dollars in economic losses annually. Border controls form the last line of defense against invasions, whereby inspectors aim to intercept and stop consignments that are contaminated with harmful alien insects. In Europe, member states depend on one another to prevent insect introductions by operating a first point of entry rule – controlling goods only when they initially enter the continent. However, ensuring consistency between border control points is difficult because there exists no optimal inspection strategy. For the first time, we developed a method to quantify the volume of agricultural trade that should be inspected for quarantine insects at border control points in Europe, based on global agricultural trade of over 100 million distinct origin-commodity-species-destination pathways. This metric was then used to evaluate the performance of existing border controls, as measured by border interception results in Europe between 2003 and 2007. Alarmingly, we found significant gaps between the trade pathways that should be inspected and actual number of interceptions. Moreover, many of the most likely introduction pathways yielded none or very few insect interceptions, because regular interceptions are only made on only a narrow range of pathways. European countries with gaps in border controls have been invaded by higher numbers of quarantine alien insect species, indicating the importance of proper inspections to prevent insect invasions. Equipped with an optimal inspection strategy based on the underlying risks of trade, authorities globally will be able to implement more effective and consistent border controls.

## Introduction

Invasive alien insects are being unintentionally moved around the world at unprecedented rates [1] as contaminants of international trade [2]–[4], impacting on ecosystems, agriculture, forestry and human health, resulting in billions of dollars in economic losses annually [5]–[7]. Europe has already been invaded by over 1,000 insect species, including some of the most invasive insects such as the Tobacco Whitefly (*Bemisia tabaci)*, the Western Corn Rootworm (*Diabrotica virgifera*) and the Colorado Potato Beetle (*Leptinotarsa decemlineata*) [8]. With ever-increasing international trade, both the number of invasions and the scale of their impacts are expected to increase [9], [10].

The international response to invasions has been driven by agreements such as the World Trade Organization Agreement on the Application of Sanitary and Phytosanitary Measures (SPS), the International Plant Protection Convention (IPPC) of the Food and Agricultural Organization of the United Nations and the Convention for Biological Diversity (CBD), with a strategic focus on prevention as the most cost-effective management method [11]–[13]. For example, the IPPC has developed a list of International Standards and Phytosanitary Measures (ISPM’s), which set out rules and recommendations for all aspects of the trade process, *e.g*. ISPM 15 “Guidelines for regulating wood packaging in international trade” which are aimed at reducing the likelihood of insect dispersal. However, there are gaps in international regulatory frameworks for the management of unintentional species movements, which includes the majority of invasive insects, because of the difficulties in evaluating the efficacy of prevention measures [14], [15].

Only plant-pest insect species are regulated in Europe. Economically harmful insects are “black-listed” and banned from entering and being moved around the continent (European Council Directive 2000/29/EC on protective measures against the introduction into the community of organisms harmful to plants or plant products and against their spread within the community). As a last line of defense against invasions from regulated insects, incoming consignments are controlled through phytosanitary inspections at Europe’s borders. Interceptions of quarantine species are entered into EPPO’s central communication database (European Council Directive 2000/29/EC). Under current legislation inspectors must check all consignments that could contain quarantine insects. Inspectors carry out inspections armed with knowledge of the Europhyt database results, together with general taxonomic and distribution data about the quarantine insects. Whilst inspectors must check all consignments that could contain quarantine insects according to the European Council Directive, exact sampling volumes and methods can vary between European member states because no optimal inspection strategy exists [16]. Moreover, making decisions regarding sampling volumes is becoming more difficult because of increasing international trade whereby inspectors only have the capacity to sample a small fraction of total imports [16]–[18]. For example of scale, only 2% of all border crossing cargo arriving at maritime ports, airports and land crossings into the US is inspected [19]. Because Europe operates a “first point of entry rule”, this can lead to border control inconsistencies regarding border inspections. Indeed member states depend on the border control efficacy of one another, as phytosanitary inspectors control goods only when they initially enter the continent.

A further concern is that there exists no method to evaluate the performance of existing border controls [14], [15], mainly because pathway management [11], [20], invasions of insects as a taxonomic group [21] and optimal detection strategies [16] have been understudied in invasion biology. Hence, thus far, border controls have only been analyzed on a stand-alone basis using interception data [22]–[24]. There is therefore a danger that inconsistencies between the border control points of Europe exist, leaving Europe highly exposed to quarantine alien insect invasions. The European Union acknowledges that a more coordinated response to insect invasions is required by its member states [14], [15], [25], with the need for pathway risk management to support risk assessment [15], [26].

Variables such as the volume and identity of goods traded, their origin and destination, can be integrated with aspects of insect biology to estimate the likelihood of unintentional insect introductions through trade [15]. Such data has been used to analyze global invasion patterns, regarding the importance of transport hubs [27] and the role of the worldwide airline network combined with climatic similarity [28] in the dispersal of alien disease vector species. Although more specific case studies have been undertaken, such as determining the dispersal risk of forest insects with specific trade pathways [29], [30] and imports of Chrysanthemum (*Dendranthema grandiflora*) cuttings by the Netherlands [16], a general analysis of Europe’s border controls against insect introductions is lacking.

We developed for the first time, a method to quantify the volume of trade that is subject to inspection in Europe, *Trade Volume to be inspected (TV),* and measured the performance of border inspections by relating TV to the actual number of insect interceptions; the *Trade Volume to be inspected Per Interception (TVPI)*. We expected that TV would be positively correlated to the number of insect interceptions, else inspection biases exist, which lead to gaps in border controls. Furthermore, we hypothesized that European countries with the weakest border controls, measured by the highest TVPI (*i.e.* the lowest frequency of interceptions per TV) are likely to have the highest levels of alien insect invasions.

## Materials and Methods

### Development of Indices: TV and TVPI

We developed a general method to quantify the volume of agricultural trade; a major invasion pathway into Europe [22], [24], that is subject to phytosanitary controls. Trade pathways consist of many components [15], of which we considered four; country of origin *o*, agricultural commodity being traded *c*, quarantine insect species *i*, and European destination country *d*, so that each pathway *o-c-i-d* can be uniquely represented. We included all quarantine insect species in Europe (*i* = 200 insect species) in our analysis, compiling their global distribution, agricultural host plants, and border interception data, together with traded value (US$) in agricultural commodities listed by the FAO (*c* = 126 commodity types) between origins (*o* = 167 origin countries) and European destinations (*d = 28* countries), totaling 117,835,200 distinct *o*-*c*-*i*-*d* pathways (Tables S1, S2, S3, S4, S5, S6, S7).

We defined the *Trade Volume to be inspected (TV)* by importing European country *d* when importing commodity *c* from origin country *o*, as the value of trade in commodity *c* (in US$), if *both* insect species *i* exists in origin country *o* and commodity *c* is a host. Therefore, if either insect *i* does not exist in origin *o*, or commodity *c* is not a potential host, then the TV of that *o-c-i-d* trade is zero. Thus TV is only positive on pathways that could potentially move quarantine insects through trade. For example, if a European country is importing maize from Argentina, then the TV of the Western Corn Rootworm (*Diabrotica virgifera*) moving on this pathway is zero because, despite maize being its primary host, the insect species does not exist in Argentina. Alternatively, the TV of the Western Corn Rootworm arriving into Europe from North America on mangos is also zero, because despite North America being within its distribution range, mango is not a host for any part of the Western Corn Rootworm’s life cycle. Therefore, rather than just using total trade volumes to relate to invasions [1], which take no account of insect species distributions or biology, TV is a more accurate approximation of the import volumes that Europe’s phytosanitary inspectors must check.

TV can be aggregated through the summation of the individual *o-c-i-d* pathways, and thus TV represents the trade volume to be inspected, weighted by the number of quarantine insect species that can be dispersed by that pathway. For example, summing TV across all pathways with origin *o* = USA enables us to quantify the total TV of all agricultural trade, weighted across all quarantine insect species, into all European countries, originating from the USA. Repeating this for all exporting countries allows us to compare the TV between world origins, and similarly, between commodities, insect species and European countries.

The performance of border controls should be measured by their output – the number of insect interceptions. We would expect that trade pathways with a higher TV would yield a higher number of insect interceptions, because phytosanitary inspectors should target those pathways that carry a higher insect contamination risk, otherwise there could be biases in controls. For example, a European country with a high TV (*i.e.* a large importer of agricultural goods, of which many could potentially be contaminated by quarantine insects) has a high level of exposure to alien insect arrivals, and hence should be expected to intercept more quarantine insects than a country with low TV. Hence, to evaluate the performance of border controls, we calculated the *Trade Volume to be inspected Per Interception (TVPI)*, calculated as the TV divided by the number of insect interceptions made on that *o-c-i-d* pathway, and interpreted as the volume of trade to be imported per insect interception made (*i.e*. the reciprocal of the interception rate per TV). High levels of TVPI indicate pathways with weak border controls (low interception rate) because few insect interceptions are made in relation to trade volumes that could be carrying quarantine insects, the TV. TVPI can thus be interpreted as a measure of the number of insects passing through border controls, or the propagule pressure. For example, the Netherlands has a TVPI = $54,623, hence they make a quarantine insect interception per $54,623 of TV, whereas Italy only makes one interception per $3,074,660 of TV (*i.e*. Italy intercepts insects far less frequently, per TV, than the Netherlands). Hence, the metric TVPI can be used to identify those pathways in Europe that yield too few insect interceptions, and require further and immediate attention by biosecurity management authorities.

We explore some of the factors that could explain variations in TVPI such as country wealth (*e.g.* rich countries can afford to invest more in border controls) and taxonomic differences between insect families and plant order (*e.g.* some insect families are easier to detect and thus more often looked for) which could influence the likelihood of insect contaminations being detected. Note that there are many other factors that potentially influence TVPI, such as the role of packaging, that we do not consider in this study.

**Figure 1:**
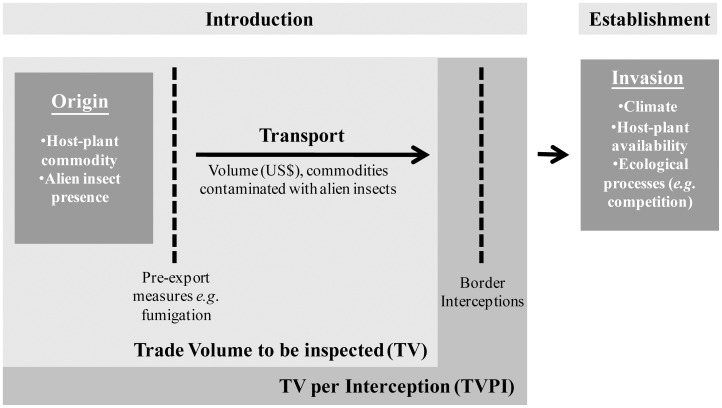
Insect dispersal through agricultural trade. We defined the *Trade Volume to be inspected (TV)* to importing European country *d* when importing commodity *c* from origin country *o*, as the value of trade in commodity *c* (in US$), if *both* insect species *i* exists in origin country *o* and commodity *c* is a host. Therefore, if either insect *i* does not exist in origin *o*, or commodity *c* is not a potential host, then the TV of that *o-c-i-d* trade is equal to zero. Thus TV is only positive on pathways that could potentially move quarantine insects through trade, and should be interpreted as a measure of the likelihood of alien insects moving through trade. We calculated the *Trade Volume to be inspected Per Interception (TVPI)* as the TV divided by the number of interceptions made per origin *o*, commodity *c*, insect species *i*, or European destination *d*. Other factors which affect the likelihood of insect dispersal, such as pre-export controls, were not included in this study since all exporters must fulfill the International Plant protection Convention (IPPC) standards and regulation. TVPI and TV do not measure other factors that determine establishment success, such as climate, host-plant availability and ecological processes.

We hypothesized that TVPI rather than TV should be related to the level of insect invasions in Europe, if border controls play a significant preventative role, because TVPI can be considered as a proxy for the propagule pressure – the number of individuals of an alien species that are introduced to the invaded region [31], which is generally a key determinant of invasion success [32]. The role of TV and TVPI within the insect dispersal process are illustrated in Figure 1.

### Data

We included all quarantine alien insect species listed by the EU Directive (European Council Directive 2000/29/EC) and the European and Mediterranean Plant Protection Organization (EPPO) as of the end of December 2010, which totalled 200 species (Table S6). Many species featured in both lists, so we used an AND/OR approach to combine the lists. All of these insects pose a serious threat for agriculture in Europe or could do so once they enter the continent and thus they are banned from entering and being moved around (European Council Directive 2000/29/EC). For each insect species, we compiled country-level data for their worldwide ranges (EPPO, Centre for Agriculture and Biosciences International (CABI) Crop Compendium, and Delivering Alien Invasive Species Inventories for Europe (DAISIE, www.europe-aliens.org)). In total, 167 non-European countries were included. Inconsistencies between the datasets were resolved using an AND/OR approach, so that the distribution ranges we used represented a maximal potential range. European countries that have eradicated a quarantine species were not included in the distribution ranges of such species. World agricultural trade data was obtained from the Food and Agriculture Organization (FAO), and we recorded import values in US$ for specific commodities originating from non-European countries, into all 28 EPPO-reporting European countries (Table S2). We included the latest available data, and averaged over the 5-year period between 2003 and 2007. We used US$ values in trade rather than weight in kg (which yielded largely the same results), for comparability of commodities that are traded in vastly different volumes. The FAO database includes a total of 548 agricultural commodity products. However, we only considered those commodity fields that are traded in natural, unprocessed and unmanufactured form, totalling 126 commodities in this study (Table S5). This included even those agricultural commodities that are not mass-produced in Europe, but that could nevertheless be host to at least one quarantine insect species. For example, bananas have a high TV in Europe because they can host several quarantine insects such as *Unaspis citri* and *Aleurocanthus woglumi,* which are polyphagous feeders and could be serious pests of *e.g*. citrus fruits in Europe.

We extracted border insect interception data from EPPO (Reporting Service) between 2003 and 2007, for each of the 200 quarantine-listed insect species. By law, European countries are required to report all interceptions of quarantine insects (European Council Directive 2000/29/EC). We recorded only those interceptions made on agricultural commodities listed in the FAO trade database, which meant excluding interceptions on “Cut flowers and branches” (2,038 interceptions) and on basil *Ocimum basilicum* (1,924 interceptions). Furthermore, we only considered interceptions of the 200 quarantine-listed species, so that each interception could be assigned to a unique *o-c-i-d* pathway. Therefore, we also excluded incomplete interception records such as “Non-European Tephritidae” (1,055 interceptions). However, the “Non-European Tephritidae” interceptions consisted primarily of interceptions made on mangos (424 interceptions) and from Thailand (460 interceptions) which if included, only strengthened our results - that interceptions on these pathway are highly numerous compared to other pathways which pose similar and higher risks. In total we included 1,148 interception records in this study, which could each be assigned to a unique *o-c-i-d* pathway, and accurately reflect the number of complete interception records made on agricultural imports in Europe.

In parallel, we compiled a database of host plant ranges for each of the quarantine insect species, aligned to commodities in our FAO commodity list, by combining data from EPPO and CABI on an AND/OR basis (Table S1). For some insect species, host-plant ranges were reduced after taking into account biological factors that limited their ability to be dispersed through trade. For example, the Hemipteran bugs *Margarodes prieskaensis, M. vitis* and *M. vredendalensis*, are associated with the common grape (*Vitis vinifera*) but grapes were not included as potential dispersal hosts because these insect species are sessile on plant roots in the soil and are very unlikely to be dispersed by fruit trade (EPPO data sheets on Quarantine Pests). In general, root-feeding insects such as Coleopteran species in the families Scarabaeidae and Chrysomelidae were included in fruit and vegetable trade since they have previously been intercepted on vegetables (EPPO data sheets on Quarantine Pests). Potato plants such as *Solanum tuberosum* are potential hosts for many of the quarantine species, however few are associated with the actual potato tubers which are usually moved in trade. Therefore, potato tubers were not considered as potential dispersal hosts for the following potato foliage-feeding insects; *Bemisia tabaci*, *Helicoverpa sp., Thrips palmi, Liriomyza sp*., *Spodoptera sp., Cacoecimorpha pronubana*, and *Tuta absoluta* (EPPO data sheets on Quarantine Pests).

Nominal Gross Domestic Product (GDP) was used as a measure of a country’s economic wealth. GDP data at current prices in US$ were obtained from the International Monetary Fund (IMF) and averaged from 2003–2007. We also tested European interception data against the importance of agriculture in GDP, hypothesizing that countries with important agricultural sectors care more about preventative border controls. We gathered importance of agriculture as a percentage of GDP from the World Factbook Central Intelligence Agency 2010 (CIA).

### Statistics

We used Pearson’s product-moment correlation coefficient *r* to test for relationships between variables such as TV, the number of interceptions and TVPI. A generalised linear model was used to test for the effects of TVPI on the level of invasion in Europe, correcting for the confounding effects of climate (latitude of capital city), climatic heterogeneity (altitudinal difference between lowest and highest point), and country area (Table S7). The number of quarantine insects established in European countries (DAISIE) was taken as dependent variable, and latitude, altitudinal difference, country area, and country TVPI were used as independent variables, all scaled to zero mean and one standard deviation [33]. Correlation between independent variables was small (all r<0.5, all variance inflation factors <2.3, Fig. S1) [34], thus ruling out collinearity. We fitted a generalised linear model to the data assuming a Poisson distribution of the dependent variable. We checked model assumptions by calculating the dispersion parameter (residual deviance/degrees of freedom), which should be around 1 [35].

## Results

**Figure 2:**
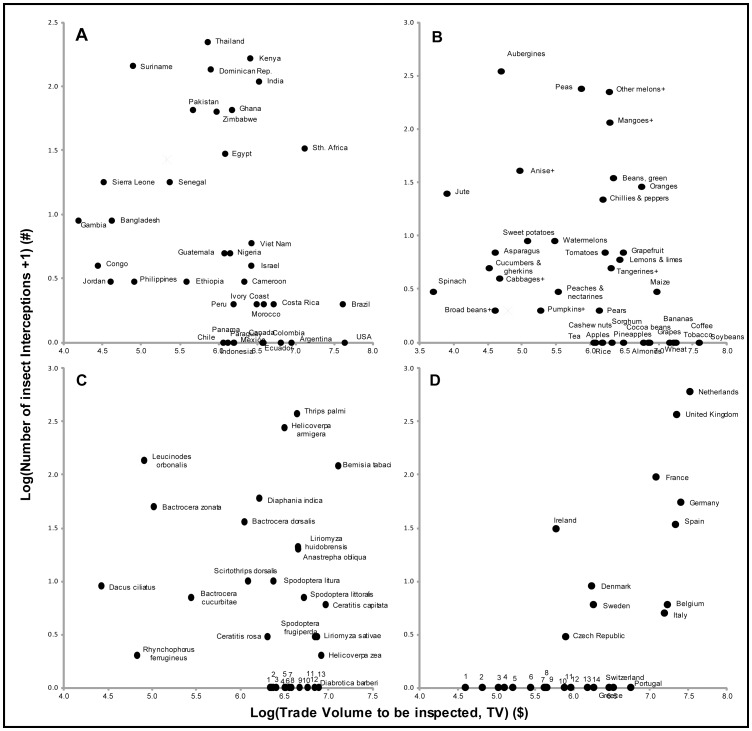
Trade volume and the number of interceptions. The relationship between the *Trade Volume to be inspected (TV)* and the number of alien insect interceptions at Europe’s borders on agricultural imports (2003–2007), by A) country of origin (r = 0.02, n = 146, p = 0.810), B) commodity type (r = −0.01, n = 126, p = 0.910), C) alien insect species (r = 0.23, n = 116, p = 0.013) and D) European importing countries (positive correlation, r = 0.73, n = 28, p = 0.00001). Only the top 25 data points by TV and number of interceptions are displayed. Notes: B) ^+^see Table S5 for full FAO commodity names. C) 1 =  *Cacoecimorpha pronubana*, 2 = *Pheletes californicus*, 3 = *Tetranychus evansi*, 4 = *Spodoptera eridania*, 5 = *Frankliniella occidentalis*, 6 = *Unaspis citri*, 7 = *Opogona sacchari*, 8 = *Rhynchophorus palmarum*, 9 = *Metamasius hemipterus*, 10 = *Liriomyza trifolii*, 11 = *Anastrepha fraterculus*, 12 = *Maconellicoccus hirsutus*, 13 = *Aleurocanthus woglumi* (see Table S3 for taxonomy). D) 1 = Luxembourg, 2 = Latvia, 3 = Estonia, 4 = Slovakia, 5 = Malta, 6 = Cyprus, 7 = Lithuania, 8 = Hungary, 9 = Slovenia, 10 = Bulgaria, 11 = Austria, 12 = Finland, 13 = Romania, 14 = Poland.

We found only weak correlations (r<0.25) between the number of insect interceptions and *Trade Volume to be inspected* (TV) by country origins (r = 0.02, n = 146, p = 0.810), traded commodities (r = −0.01, n = 126, p = 0.910) and insect species (r = 0.23, n = 116, p = 0.013) (Figs. 2A–C), indicating biases in Europe’s border controls. Country of origin control bias by economic wealth, measured by Gross Domestic Product (GDP) was suspected because Asian and African countries dominated the interception database (73% of interceptions) despite the highest TV coming from the Americas (69% of total TV) (Fig. 2A, Table S4). However, we found no significant correlation between the number of insect interceptions and origin GDP (r = −0.10, n = 123, p = 0.270).

Commodity and insect species showed control bias because only a small fraction of pathways yielded regular interceptions. Most frequently intercepted were infected aubergines, green peas, “other melons” and “mangoes, mangosteens and guavas” and insects such as Palm thrips (*Thrips palmi)*, the Cotton Bollworm (*Helicoverpa armigera)*, the Eggplant Borer (*Leucinodes orbonalis)* and the Tobacco Whitefly (*Bemisia tabaci)*, despite the broad spectrum of TV (Figs. 2B & 2C, Tables S5–S6). Moreover, the vast majority of commodities (76%) and quarantine insect species (81%) yielded zero interceptions. Taxonomy was tested as an explanation of plant commodity and insect species control bias, to account for factors affecting inspection efficacy, such as variations in physical properties between commodities and life history traits of quarantine insects. However, we found good correlations between the number of interceptions and TV aggregated by plant order (r = 0.58, n = 21, p = 0.006) and insect family (r = 0.58, n = 31, p = 0.001), indicating that interceptions were not biased according to particular taxonomic groups. By contrast, we found interception bias between insect species and plant commodities within taxonomic groups. Bias exists within 6 out of 7 plant orders – Asparagales (r = 0.18, n = 6, p = 0.730), Ericales (r = 0.18, n = 7, p = 0.700), Fabales (r = −0.07, n = 14, p = 0.810), Rosales (r = −0.04, n = 15, p = 0.89), Sapindales (r = 0.19, n = 8, p = 0.650) and Solanales (r = 0.22, n = 7, p = 0.640), and within all insect families with n>5– Curculionidae (r = −0.10, n = 21, p = 0.670), Noctuidae (r = −0.15, n = 7, p = 0.750), Tephritidae (r = 0.11, n = 36, p = 0.520) and Tortricidae (r = −0.11, n = 11, p = 0.750).

Overall, we found that the insect species with the highest TVPI are the Northern Corn Rootworm (*Diabrotica barberi)*, the Citrus Blackfly (*Aleurocanthus woglumi*) and the Pink Mealybug (*Maconellicoccus hirsutus*). These are the insect species for which the highest trade volume (TV) needs to arrive per interception, assuming current practice, thus these are the species that are most likely to slip past border controls. The most likely pathways of entry measured by TVPI are on agricultural imports from the Americas, especially from both the U.S.A. and Brazil, and on commodities such as soybeans, tobacco, coffee, bananas and wheat (Figs. 2A–C, Tables S4, S5, S6).

**Figure 3:**
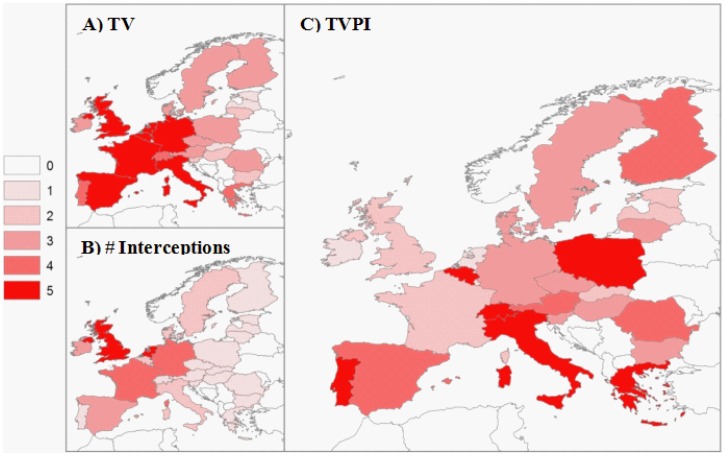
European country import volumes and interceptions. Heat maps showing A) *Trade Volume to be inspected (TV)*, B) the number of border insect interceptions (+1) and C) the *Trade Volume to be inspected Per Interception (TVPI)* in Europe. In each map, log values were used and split linearly into a 5 point scale, with 1 representing the smallest 20% of the log value range, through to 5 which represents the highest 20% of the log value range. In all maps, white countries scaled 0, were not included in this study.

We also analyzed the border controls of European countries and found that alien insects are intercepted in proportion to TV (r = 0.73, n = 28, p = 0.00001) (Fig. 2D). However, high TVPI pathways exist because the majority of member states (17 out of 28) recorded not a single insect interception, despite all having some level of TV (Fig. 3, Table S7). We found no control bias by testing the number of insect interceptions against the capacity of European countries to implement border controls (countries’ wealth estimated by GDP) (r = 0.21, n = 28, p = 0.280) and the importance of agriculture (as a % of GDP) (r = −0.21, n = 28, p = 0.280).

**Figure 4:**
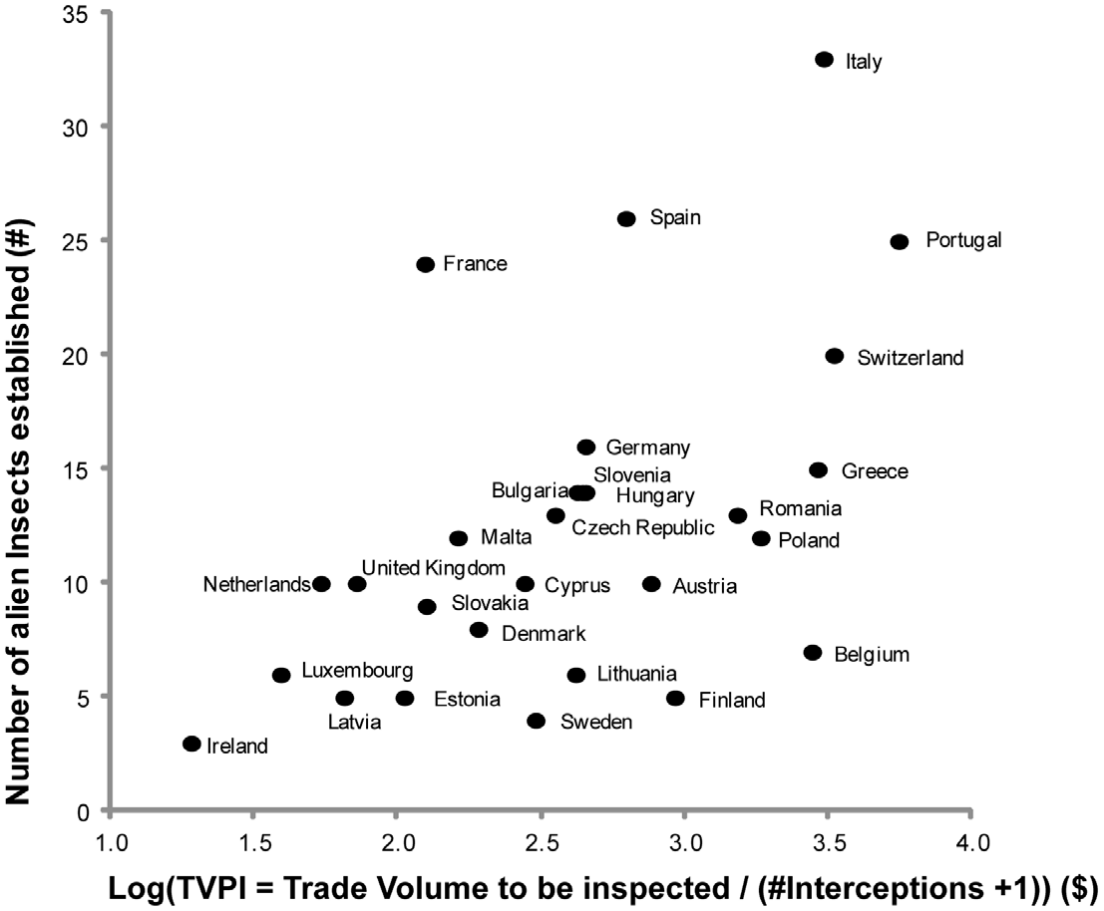
Border controls and the level of invasion. *Trade Volume to be inspected Per Interception (TVPI)* and the level of invasion in Europe. We found a positive correlation between the TVPI (2003–2007) and the number of quarantine insects that have established per European country (r = 0.54, n = 28, p = 0.003). The effect of TVPI on invasion is still significant (p = 0.0398) if we include climatic factors such as capital city latitude (p = 0.00002), country area (p = 0.0504) and altitudinal range (p = 0.0027) as proxies of climatic range for each reporting country (generalised linear model with Poisson errors) (Fig. S1).

The number of alien species that have already established in each country (Table S3) positively correlated to the TVPI (r = 0.54, n = 28, p = 0.003) (Fig. 4), our proxy for propagule pressure, rather than to TV (r = 0.36, n = 28, p = 0.06), indicating that border controls play a role in preventing insect invasions. This is particularly evident in the Netherlands and the UK, who are among the countries with the highest interception rates (lowest TVPI), and have suffered relatively low levels of insect invasions, despite having the highest TV levels. The effect of TVPI on invasion in European countries remains significant if we include other confounding factors that might explain the level of invasion, e.g. the general climate (as latitude), area and altitudinal range for each reporting country (generalised linear model with Poisson errors: p = 0.0398) (Table S7, Fig S1). Alarmingly, we found that countries with the most favorable climatic conditions for alien insect invasions (low latitude, high altitudinal ranges) – Portugal, Switzerland, Italy and Greece, also have the highest TVPI because of weak border controls (Fig. 3).

## Discussion

Europe is highly exposed to insect introductions through agricultural trade, with control gaps, indicated by high TVPI, existing across a wide range of pathways. Inconsistencies were not surprising given the absence of an optimisation strategy on which border control authorities should base sampling efforts. Although control bias between pathways could not be explained, they are difficult to justify because all types of agricultural commodities from all country origins are regulated for all quarantine-listed insects under the IPPC, and phytosanitary certificates are required to confirm that international standards have been reached (IPPC; European Council Directive 2000/29/EC).

Preventative border controls therefore have a key role to play, especially since 145 out of 200 quarantine-listed species have not yet established in Europe (Table S3). Moreover, TVPI was significantly related to the number of insect invasions, even though only a small proportion of total incoming goods are likely to be inspected [19]. Hence, phytosanitary inspections likely reduce the propagule pressure by acting as a deterrent for exporting contaminated goods.

In particular, the Americas were under-represented in Europe’s interception databases according to TVPI. This is worrying because the Americas are known to be a major source of insect invasions, contributing 33% of all insect species invasions in Europe to date (Roques *et al.* 2010). Furthermore, a study of cargo aircraft from Central America showed that 23% of flights were infested with live hitchhiking insects [36]. Insect invasions of American origin include some of the worst pests (DAISIE), such as the Western Corn Rootworm (*Diabrotica virgifera)*, which is associated with trade from North America [37] and has a European distribution centered around international transport hubs (EPPO). Such transport hubs are stepping stones for invasions, being both more likely to be invaded and therefore likely to enhance the further spread of invasions to secondary locations [27]. Hence with free intra-European trade, quarantine species that enter Europe at major transport hubs such as international airports and maritime ports, pose significant invasion risks to the rest of Europe. For example, we identified the Northern Corn Rootworm (*Diabrotica barberi)*, a close relative of the Western Corn Rootworm, as one of the most likely European arrivals under existing control practices (Fig. 2C), with a high TV, but no reported interceptions. Although GDP was not found to significantly explain the country of origin control bias, it is likely that the perception of risk and political relationships (*e.g.* historical relationships, trade agreements and trust) plays some role in inspection sampling efforts.

Commodities that are imported on a large-scale, such as soybeans, tobacco, coffee, bananas and wheat (Fig. 2B), and their quarantine insect associates, represent the highest TVPI in Europe. Taxonomic differences between commodities and insects did not explain the control biases, but other practical and physical limitations may influence control efficacy. For example, adequately inspecting large-volume shipments of commodities such as soybeans, for endophytic insects, poses significant logistical challenges to inspectors because of both difficulties of scale and the hidden nature of many insect contaminations. Furthermore, the state in which import commodities arrive in Europe, such as the level by which they have been part-processed and the accessibility of different packaging types, are all likely to influence inspection efficacy. In other studies about the role of transport in facilitating insect dispersal, a wide range of commodity pathways and a broad spectrum of insect species have been recorded [23], [36], [38], suggesting that Europe’s interception database consists of a too narrow pathway focus.

The interception biases we found in Europe might be an indication that inspection sampling is influenced by historic interception database records, which are used for prioritization in the absence of a general method to quantify the true underlying risks. This has a compounding effect – pathways that yield insect interceptions now are thus more likely to be targeted by future inspections. In turn, this process narrows the number of pathways that are subject to border inspection. This is especially evident when considering interceptions by either commodity type or insect species, which are both dominated by just 4 types/species (Fig. 2B & 2C) and show inspection bias between taxonomically related groups. Interception databases should only be used as a guide for inspection sampling if total inspection efforts are recorded which would allow the calculation of interception rates [22]; otherwise we recommend that sampling efforts should be distributed in proportion to TV.

Gaps in border controls measured by TVPI, caused by biased interception data not reflecting fairly the TV, were shown to significantly correlate to high levels of insect invasions in Europe. Biosecurity authorities should be aware that targeting inspections to better control pathways with high TVPI could help to prevent insect invasions, and should be given immediate attention and further investigation to determine if high TVPI is justifiable. Firstly, high TVPI pathways should be tested for bias by obtaining phytosanitary inspector sampling effort statistics, rather than just positive interception records [22]. Evidence of low sampling effort can be addressed by increasing inspection effort according to TV, or increasing and improving inspection resources *i.e.* an increase in inspector numbers and training. For pathways with high TVPI despite high sampling effort, novel insect detection techniques could be employed at border control points to test the efficacy of phytosanitary inspection as a general insect detection measure. For example, a series of insect traps aimed at quarantine insect species could be installed at major international transport hubs in Europe to complement phytosanitary inspections.

However, many invasive insect species that were not previously considered as quarantine have established in Europe [8]. Current border controls only target the quarantine-listed insect species in plant protection. Hence using interception data, we can only measure the efficacy of existing control measures, in relation to the 200 quarantine species that they were set-up to intercept. Therefore, actual gaps in border controls are higher than indicated by TVPI because many non-quarantine-listed insects also have invasion potential and are not controlled by border authorities. In comparison, the world-leading biosecurity authorities of New Zealand [39] and Australia [40] take a more stringent white-list approach to quarantine, whereby border controls target the interception of all incoming alien insect species. An important issue for European authorities is whether a black-list of known invasive insects, as analysed in this study, or broader white-list approach make a more effective border control strategy.

Under current Europe-wide legislation, it is crucial that member states maintain coordinated and consistent border control strategies. This can be achieved by basing sampling efforts according to the underlying TV, reducing biases and hence TVPI which correlates to invasion. Optimising sampling efforts [16] can reduce the environmental and economic impacts of insect invasions both now [5]–[7] and in the future [10]. Social and political complications that arise through applying such measures could be overcome if Europe established a central authority to integrate regulation with management response [13]. TVPI and TV could also be quantified more widely for any set of origins, destinations and commodities traded, such as forestry products [1] and ornamental plants [22], [24], and adapted to include any insect species list, including disease vectors as well as plant pests, to assist the risk assessment of alien insect introductions worldwide.

## Supporting Information

Figure S1 **Generalised linear model results.** We used a generalised linear model to test for the effects of TVPI on the level of invasion in Europe, correcting for the confounding effects of climate (latitude of capital city), climatic heterogeneity (altitudinal difference between lowest and highest point), and country area. The number of quarantine insects established in European countries (DAISIE) was taken as dependent variable, and latitude, altitudinal difference, country area, and country TVPI were used as independent variables, all scaled to zero mean and one standard deviation [33]. Correlation between independent variables was small (all r<0.5, all variance inflation factors <2.3, Fig. S1) [34], thus ruling out collinearity. We fitted a generalised linear model to the data assuming a Poisson distribution of the dependent variable. We checked model assumptions by calculating the dispersion parameter (residual deviance/degrees of freedom), which should be around 1 [35].
(PDF)

Table S1 **Insect species matrix origin-commodity.** Country-level distribution range and host-plant associations (FAO commodities) for all 200 quarantine listed insect species in Europe.
(XLSX)

Table S2 **Trade Volume to be inspected (TV) in $th, per European Country from world (including Europe).**
(XLSX)

Table S3 **European distribution range of quarantine insect species (DAISIE and EPPO).**
(XLSX)

Table S4 **Country origin of agricultural imports by Europe for the 5-year period 2003 to 2007.** For each non-European country, data shown are; the total value of agricultural exports to Europe (US$th, FAO), the Trade Volume to be inspected (TV), the number of quarantine alien insect interceptions (EPPO), the Trade Volume to be inspected Per Interception (TVPI) (ranked), and Nominal GDP ($, IMF).
(PDF)

Table S5 **Agricultural commodity imports by Europe for the 5-year period 2003 to 2007.** For each FAO commodity, data shown are; the total value of exports to Europe (US$th), the Trade Volume to be inspected (TV), the number of quarantine alien insect interceptions (EPPO), and the Trade Volume to be inspected Per Interception (TVPI) (ranked).
(PDF)

Table S6 **Control of quarantine alien insects in Europe for the 5-year period 2003 to 2007.** For each quarantine alien insect, data shown are; EU1 if the species is listed in the EU Directive 2000/29/EC, EPPO2 if the spedies is listed in the EPPO quarantine lists, the Trade Volume to be inspected (TV), the number of quarantine interceptions (EPPO), and the Trade Volume to be inspected Per Interception (TVPI) (ranked).
(PDF)

Table S7 **European country insect invasions data for the 5-year period 2003 to 2007.** For each European country, data shown are; the total value of agricultural imports (US$th, FAO), the Trade Volume to be inspected (TV), the number of quarantine alien insect interceptions (EPPO), the Trade Volume to be inspected Per Interception (TVPI) (ranked), the number of quarantine listed alien insects that have established (DAISIE, EPPO), the Nominal GDP ($, IMF), Agriculture as a % of GDP, Capital city latitude (degrees), Country area (km2)and altitudinal range (m).
(PDF)
