# Supplementary material for: Gaps in Border Controls Are Related to Quarantine Alien Insect Invasions in Europe
Source: PLoS One. 2012 Oct 24;7(10):e47689. doi: 10.1371/journal.pone.0047689 (PMC3480426; doi:10.1371/journal.pone.0047689)
Supplement: Figure S1 — Generalised linear model results. We used a generalised linear model to test for the effects of TVPI on the level of invasion in Europe, correcting for the confounding effects of climate (latitude of capital city), climatic heterogeneity (altitudinal difference between lowest and highest point), and country area. The number of quarantine insects established in European countries (DAISIE) was taken as dependent variable, and latitude, altitudinal difference, country area, and country TVPI were used as independent variables, all scaled to zero mean and one standard deviation [33]. Correlation between independent variables was small (all r<0.5, all variance inflation factors <2.3, Fig. S1) [34], thus ruling out collinearity. We fitted a generalised linear model to the data assuming a Poisson distribution of the dependent variable. We checked model assumptions by calculating the dispersion parameter (residual deviance/degrees of freedom), which should be around 1 [35]. (PDF) [file pone.0047689.s001.pdf]

	Estimate	S.E.	z value	p-value
Intercept	2.40	0.060	39.91	< 0.001
TVPI	0.11	0.054	2.06	0.0398
Latitude	0.25	0.067	-3.71	0.0002
Area	0.13	0.066	1.96	0.0504
Altitudinal range	0.23	0.077	3.00	0.0027

Distribution of residuals

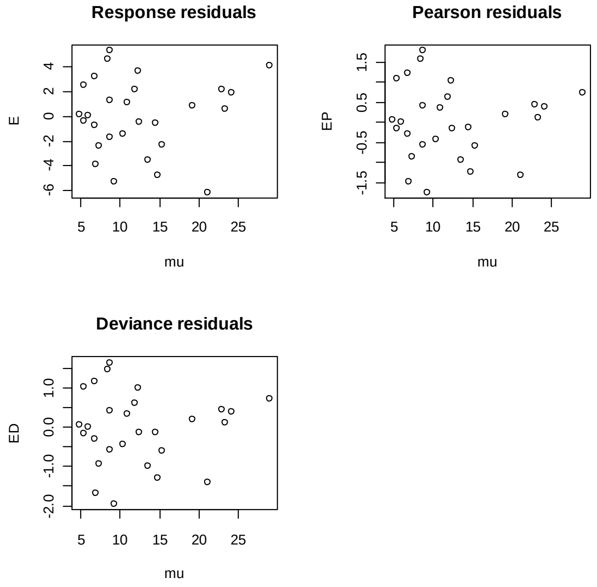

Fig S1. Generalised linear model assuming a Poisson distribution of the response variable on the relationship between a country's invasion level (= number of quarantine insects established in the country) and the TVPI of these insects, the latitude of the country's capital (as a proxy for the climate, from warm and dry Mediterranean countries in the south to cool and wet northern countries), the country's area (larger countries are expected to have more species), and the country's altitudinal range (as a proxy for the climatic variation within countries). All independent variables were scaled to zero mean and 1 standard deviation before analysis. Null deviance is 110.1 on 27 degrees of freedom, the residual deviance is 22.96 on 23 degrees of freedom. About 79% of the deviance is explained by the model (null deviance – residual deviance)/null deviance. The dispersion parameter was assumed to be 1, which is a good approximation of the model fit (residual deviance/degrees of freedom =

22.96/23=0.998). The model shows that a country's invasion level is positively related to TVPI, area (not quite significantly) and altitudinal range, and negatively to its latitude. There were no indications of collinearity between variables (Pearson correlation coefficients all << 0.7 and variance inflation factors all << 3 [34]). There was no pattern in the distribution of residuals visible, thus supporting the appropriateness of the model fit.
