# Supplementary material for: Gaps in Border Controls Are Related to Quarantine Alien Insect Invasions in Europe
Source: PLoS One. 2012 Oct 24;7(10):e47689. doi: 10.1371/journal.pone.0047689 (PMC3480426; doi:10.1371/journal.pone.0047689)
Supplement: Table S4 — Country origin of agricultural imports by Europe for the 5-year period 2003 to 2007. For each non-European country, data shown are; the total value of agricultural exports to Europe (US$th, FAO), the Trade Volume to be inspected (TV), the number of quarantine alien insect interceptions (EPPO), the Trade Volume to be inspected Per Interception (TVPI) (ranked), and Nominal GDP ($, IMF). (PDF) [file pone.0047689.s005.pdf]

Table S4. Country origin of agricultural imports by Europe for the 5-year period 2003 to 2007. For each non-European country, data shown are; the total value of agricultural exports to Europe (US$th, FAO), the Trade Volume to be inspected (TV), the number of quarantine alien insect interceptions (EPPO), the Trade Volume to be inspected Per Interception (TVPI) (ranked), and Nominal GDP ($, IMF).

Country	Continent	Agr. Exports Europe, $th 03-07	TV $th	#alien insect interceptions 03-07	TVPI $th	Nominal GDP, $ 03-07
United States of America	North America	4'182'838	42'633'839	0	**42'633'839**	42'571'159
Brazil	SC America	5'915'677	40'271'643	1	**20'135'821**	4'955'055
Argentina	SC America	1'225'807	8'715'420	0	**8'715'420**	4'863'177
Colombia	SC America	1'276'298	6'344'349	0	**6'344'349**	3'399'121
Ecuador	SC America	997'984	3'781'055	0	**3'781'055**	2'781'606
Canada	North America	1'074'825	3'664'491	0	**3'664'491**	35'262'906
Costa Rica	SC America	1'187'561	5'115'633	1	**2'557'817**	4'902'767
Morocco	Africa	817'225	3'815'126	1	**1'907'563**	2'025'547
Paraguay	SC America	302'406	1'573'722	0	**1'573'722**	1'418'230
Panama	SC America	318'304	1'566'518	0	**1'566'518**	4'909'914
Mexico	SC America	248'253	1'553'421	0	**1'553'421**	8'209'584
Côte d'Ivoire	Africa	1'339'853	3'084'250	1	**1'542'125**	870'858
Indonesia	Asia	364'544	1'307'436	0	**1'307'436**	1'427'782
Chile	SC America	802'527	1'139'387	0	**1'139'387**	7'361'469
Honduras	SC America	298'096	1'006'989	0	**1'006'989**	1'443'809
Russian Federation	Asia	278'224	798'656	0	**798'656**	5'692'290
Peru	SC America	483'367	1'549'716	1	**774'858**	2'995'499
Cameroon	Africa	491'598	2'131'749	2	**710'583**	941'906
Israel	Asia	605'641	2'627'398	3	**656'849**	20'666'092
Australia	Oceania	126'067	558'575	0	**558'575**	35'578'433
Malawi	Africa	191'321	529'092	0	**529'092**	222'067
Viet Nam	Asia	718'014	2'640'000	5	**440'000**	647'829
Uruguay	SC America	167'299	796'844	1	**398'422**	5'256'941
South Africa	Africa	1'163'913	12'914'827	32	**391'358**	5'026'507
Sri Lanka	Asia	123'557	387'714	0	**387'714**	1'268'384
Uganda	Africa	177'061	659'610	1	**329'805**	315'436
Papua New Guinea	Oceania	85'789	296'311	0	**296'311**	847'662
Nigeria	Africa	352'039	1'397'474	4	**279'495**	840'543
Tunisia	Africa	102'431	265'748	0	**265'748**	3'269'691
Belize	SC America	50'547	255'311	0	**255'311**	3'855'652
El Salvador	SC America	84'893	254'696	0	**254'696**	3'074'607
New Zealand	Oceania	477'044	250'926	0	**250'926**	25'572'932
Nicaragua	SC America	78'080	240'115	0	**240'115**	947'066
Guatemala	SC America	203'418	1'177'747	4	**235'549**	2'153'845
Venezuela (Bolivarian Rep. of)	SC America	36'878	198'719	0	**198'719**	5'622'827
Mozambique	Africa	58'294	171'909	0	**171'909**	329'185
Syrian Arab Republic	Asia	50'014	161'055	0	**161'055**	1'618'681
Swaziland	Africa	16'953	149'713	0	**149'713**	2'421'102
Cuba	SC America	24'690	146'724	0	**146'724**	-
Ethiopia	Africa	187'553	380'237	2	**126'746**	174'058
Saint Lucia	SC America	27'736	110'494	0	**110'494**	-
Togo	Africa	86'751	173'659	1	**86'830**	339'668
Saint Vincent & the Grenadines	SC America	14'894	74'209	0	**74'209**	-
Burundi	Africa	36'352	72'541	0	**72'541**	105'089
Guinea	Africa	34'494	70'662	0	**70'662**	370'679
Iran (Islamic Republic of)	Asia	212'229	64'383	0	**64'383**	2'858'702
Rwanda	Africa	32'346	63'210	0	**63'210**	294'086
Kazakhstan	Asia	62'335	61'520	0	**61'520**	4'122'165
Dominica	SC America	9'629	56'220	0	**56'220**	4'180'862
Lao People's Democratic Rep.	Asia	12'996	51'655	0	**51'655**	503'912
Namibia	Africa	25'150	50'049	0	**50'049**	3'620'877
Jamaica	Africa	22'481	99'067	1	**49'534**	4'183'008
Lebanon	Africa	15'545	48'101	0	**48'101**	5'936'861
Algeria	Africa	21'316	46'623	0	**46'623**	3'055'781
Egypt	Africa	347'464	1'204'932	29	**40'164**	1'383'542
Guyana	SC America	37'179	37'600	0	**37'600**	1'832'026
Japan	Asia	9'074	35'339	0	**35'339**	34'648'865
India	Asia	811'234	3'309'562	109	**30'087**	731'876

Country	Continent	Agr. Exports Europe, $th 03-07	TV $th	#alien insect interceptions 03-07	TVPI $th	Nominal GDP, $ 03-07
Philippines	Asia	17'621	80'064	2	**26'688**	1'229'518
Mali	Africa	5'437	26'239	0	**26'239**	446'431
Republic of Korea	Asia	10'664	23'051	0	**23'051**	-
Burkina Faso	Africa	7'971	45'746	1	**22'873**	416'550
Madagascar	Africa	64'170	22'847	0	**22'847**	296'445
Ghana	Africa	750'180	1'477'080	65	**22'380**	522'440
Bolivia (Plurinational State of)	SC America	15'826	20'637	0	**20'637**	1'083'332
Iraq	Asia	5'125	20'405	0	**20'405**	1'392'255
Malaysia	Asia	8'724	38'513	1	**19'256**	5'508'802
Kenya	Africa	462'082	2'544'339	166	**15'236**	611'031
Zimbabwe	Africa	167'212	932'302	63	**14'567**	429'019
Jordan	Asia	9'456	39'520	2	**13'173**	2'456'125
United Arab Emirates	Asia	9'656	12'883	0	**12'883**	34'756'117
Senegal	Africa	32'047	230'362	17	**12'798**	756'324
Singapore	Asia	5'024	12'350	0	**12'350**	29'217'585
Haiti	SC America	4'765	11'523	0	**11'523**	459'013
Saudi Arabia	Asia	2'270	10'830	0	**10'830**	13'090'174
Sao Tome and Principe	Africa	5'259	10'490	0	**10'490**	-
Bahamas	SC America	1'830	8'425	0	**8'425**	-
Myanmar	Asia	4'812	8'402	0	**8'402**	242'891
Sudan	Africa	7'746	16'334	1	**8'167**	838'250
Zambia	Africa	50'056	211'868	26	**7'847**	700'249
Pakistan	Asia	76'937	461'157	65	**6'987**	733'784
Congo	Africa	13'504	27'002	3	**6'750**	1'791'527
Democratic Rep. Congo	Africa	13'363	6'671	0	**6'671**	131'950
China, Hong Kong SAR	Asia	1'555	5'937	0	**5'937**	-
Dominican Republic	SC America	171'836	783'678	136	**5'720**	3'343'070
Liberia	Africa	2'787	5'621	0	**5'621**	157'417
Benin	Africa	2'474	4'974	0	**4'974**	529'055
Bangladesh	Asia	14'682	40'919	8	**4'547**	409'426
Trinidad and Tobago	SC America	2'183	4'443	0	**4'443**	-
Central African Republic	Africa	1'822	3'615	0	**3'615**	337'055
Mauritius	Africa	1'852	3'489	0	**3'489**	5'200'123
Thailand	Asia	172'729	715'728	223	**3'195**	2'869'878
Niger	Africa	647	1'982	0	**1'982**	266'855
Kyrgyzstan	Asia	4'121	1'971	0	**1'971**	513'086
Grenada	SC America	7'172	1'969	0	**1'969**	5'218'437
Uzbekistan	Asia	1'686	1'872	0	**1'872**	570'276
Sierra Leone	Africa	15'961	32'097	17	**1'783**	247'830
Angola	Africa	577	1'725	0	**1'725**	2'163'672
Gambia	Africa	3'008	15'154	8	**1'684**	426'290
Afghanistan	Asia	1'411	1'574	0	**1'574**	260'246
Yemen	Asia	821	1'507	0	**1'507**	786'119
Antigua and Barbuda	SC America	778	1'318	0	**1'318**	11'292'209
Vanuatu	Oceania	521	1'008	0	**1'008**	1'924'805
Nepal	Asia	656	993	0	**993**	306'364
Cambodia	Asia	713	822	0	**822**	470'109
Guam	Oceania	172	800	0	**800**	-
Suriname	SC America	20'272	76'878	145	**527**	3'641'682
Mauritania	Africa	269	509	0	**509**	712'652
Oman	Asia	78	486	0	**486**	11'818'342
New Caledonia	Oceania	344	353	0	**353**	-
Libyan Arab Jamahiriya	Africa	119	352	0	**352**	7'894'435
Turks and Caicos Islands	SC America	324	320	0	**320**	-
Tonga	Oceania	169	281	0	**281**	2'652'381
British Virgin Islands	SC America	469	277	0	**277**	-
Guinea-Bissau	Africa	398	275	0	**275**	391'934
Gabon	Africa	126	229	0	**229**	6'261'531
Cayman Islands	SC America	408	202	0	**202**	-
Barbados	SC America	29	195	0	**195**	12'532'618
Saint Kitts and Nevis	SC America	91	168	0	**168**	-
Kiribati	Oceania	140	144	0	**144**	1'133'366
Bahrain	Asia	134	141	0	**141**	18'595'501
Seychelles	Africa	114	121	0	**121**	10'900'356
Cape Verde	Africa	60	114	0	**114**	2'152'481
Brunei Darussalam	Asia	22	69	0	**69**	25'581'556

Country	Continent	Agr. Exports Europe, $th 03-07	TV $th	#alien insect interceptions 03-07	TVPI $th	Nominal GDP, $ 03-07
Solomon Islands	Asia	28	68	0	**68**	913'016
Somalia	Africa	27	64	0	**64**	-
Tajikistan	Asia	45	59	0	**59**	360'965
Netherlands Antilles	SC America	1'325	56	0	**56**	-
Saint Helena	Africa	27	53	0	**53**	-
Eritrea	Africa	27	34	0	**34**	251'236
Marshall Islands	Oceania	29	29	0	**29**	-
Lesotho	Africa	19	26	0	**26**	555'001
EQUATORIAL GUINEA	Africa	4'346	25	0	**25**	6'668'946
Kuwait	Asia	19	25	0	**25**	26'788'608
Bermuda	North America	156	20	0	**20**	-
Maldives	Asia	20	19	0	**19**	2'836'786
Bhutan	Asia	18	18	0	**18**	1'278'264
Samoa	Oceania	2	11	0	**11**	2'340'122
Democratic People's Rep. Korea	Asia	10	10	0	**10**	17'478'132
Comoros	Africa	7'707	9	0	**9**	632'957
French Polynesia	Oceania	1'417	9	0	**9**	-
Chad	Africa	11	8	0	**8**	578'306
Cook Islands	Oceania	4	6	0	**6**	-
Fiji	Asia	12	2	0	**2**	3'428'883
Qatar	Asia	2	1	0	**1**	-
Turkmenistan	Asia	7	1	0	**1**	-
Botswana	Africa	0	0	0	**0**	-
Unspecified	-	55'415	0	0	**0**	-
United Republic of Tanzania	Africa	141'862	0	0	**0**	-
Tuvalu	Oceania	0	0	0	**0**	-
Timor-Leste	Asia	848	0	0	**0**	-
TANZANIA	Africa	0	0	15	**0**	-
TAIWAN	Asia	0	0	0	**0**	-
Saint Pierre and Miquelon	North America	0	0	0	**0**	-
PUERTO RICO	SC America	0	0	0	**0**	-
Occupied Palestinian Territory	Asia	2'450	0	0	**0**	-
Niue	Oceania	0	0	0	**0**	-
Nauru	Oceania	5	0	0	**0**	-
Mongolia	Asia	1'067	0	0	**0**	-
Mayotte	Africa	255	0	0	**0**	-
Greenland	North America	13'311	0	0	**0**	-
FRENCH GUIANA	SC America	0	0	1	**0**	-
Falkland Islands (Malvinas)	SC America	13	0	0	**0**	-
Djibouti	Africa	1'141	0	0	**0**	-
China, Macao SAR	Asia	35	0	1	**0**	-
Aruba	SC America	886	0	0	**0**	-
American Samoa	Oceania	142	0	0	**0**	-
**World Total**		**30'924'242**	**170'157'879**	**1'165**	**145'933**	**-**
