# Supplementary material for: Gaps in Border Controls Are Related to Quarantine Alien Insect Invasions in Europe
Source: PLoS One. 2012 Oct 24;7(10):e47689. doi: 10.1371/journal.pone.0047689 (PMC3480426; doi:10.1371/journal.pone.0047689)
Supplement: Table S5 — Agricultural commodity imports by Europe for the 5-year period 2003 to 2007. For each FAO commodity, data shown are; the total value of exports to Europe (US$th), the Trade Volume to be inspected (TV), the number of quarantine alien insect interceptions (EPPO), and the Trade Volume to be inspected Per Interception (TVPI) (ranked). (PDF) [file pone.0047689.s006.pdf]

**Table S5.** Agricultural commodity imports by Europe for the 5-year period 2003 to 2007. For each FAO commodity, data shown are; the total value of exports to Europe (US$th), the Trade Volume to be inspected (TV), the number of quarantine alien insect interceptions (EPPO), and the Trade Volume to be inspected Per Interception (TVPI) (ranked).

| **Commodity** | **Plant Order** | **Agr. Exports Europe, $th 03-07** | **TV $th** | **#alien insect interceptions 03-07** | **TVPI $th** |
|---|---|---|---|---|---|
| Soybeans | Fabales | 4'950'150 | 39'343'233 | 0 | **39'343'233** |
| Tobacco, unmanufactured | Solanales | 3'572'099 | 17'990'832 | 0 | **17'990'832** |
| Coffee, green | Gentianales | 5'081'574 | 16'651'889 | 0 | **16'651'889** |
| Bananas | Zingiberales | 4'805'542 | 14'556'996 | 0 | **14'556'996** |
| Wheat | Poales | 5'187'496 | 7'429'482 | 0 | **7'429'482** |
| Almonds Shelled | Rosales | 1'400'920 | 7'118'478 | 0 | **7'118'478** |
| Grapes | Vitales | 2'400'107 | 7'097'462 | 0 | **7'097'462** |
| Cocoa beans | Malvales | 2'945'590 | 6'035'139 | 0 | **6'035'139** |
| Maize | Poales | 3'620'419 | 9'455'627 | 2 | **3'151'876** |
| Apples | Rosales | 2'538'251 | 3'077'694 | 0 | **3'077'694** |
| Pineapples | Poales | 977'738 | 2'086'091 | 0 | **2'086'091** |
| Sorghum | Poales | 245'873 | 1'519'732 | 0 | **1'519'732** |
| Rice Husked | Poales | 552'206 | 1'497'134 | 0 | **1'497'134** |
| Tea | Ericales | 873'259 | 1'200'492 | 0 | **1'200'492** |
| Cashew Nuts Shelled | Sapindales | 446'809 | 1'135'231 | 0 | **1'135'231** |
| Avocados | Laurales | 463'418 | 960'069 | 0 | **960'069** |
| Sunflower seed | Asterales | 811'743 | 870'614 | 0 | **870'614** |
| Plums and sloes | Rosales | 351'210 | 743'137 | 0 | **743'137** |
| Pears | Rosales | 953'046 | 1'360'868 | 1 | **680'434** |
| Walnuts, with shell | Fagales | 152'197 | 661'593 | 0 | **661'593** |
| Chick peas | Fabales | 132'413 | 556'614 | 0 | **556'614** |
| Walnuts Shelled | Fagales | 257'943 | 517'688 | 0 | **517'688** |
| Lemons and limes | Sapindales | 802'264 | 2'715'689 | 5 | **452'615** |
| Grapefruit (inc. pomelos) | Sapindales | 470'202 | 3'054'155 | 6 | **436'308** |
| Cherries | Rosales | 368'524 | 410'722 | 0 | **410'722** |
| Tangerines, mandarins, clem. | Sapindales | 1'683'692 | 2'023'188 | 4 | **404'638** |
| Dates | Arecales | 189'378 | 393'772 | 0 | **393'772** |
| Groundnuts, with shell | Fabales | 64'944 | 389'789 | 0 | **389'789** |
| Lentils | Fabales | 105'596 | 385'116 | 0 | **385'116** |
| Maize, green | Poales | 84'892 | 347'259 | 0 | **347'259** |
| Tomatoes | Solanales | 3'250'344 | 1'653'433 | 6 | **236'205** |
| Rice Broken | Poales | 157'380 | 197'792 | 0 | **197'792** |
| Oranges | Sapindales | 1'940'473 | 5'657'254 | 28 | **195'078** |
| Strawberries | Rosales | 910'495 | 170'293 | 0 | **170'293** |
| Papayas | Brassicales | 87'850 | 138'450 | 0 | **138'450** |
| Carobs | Fabales | 65'669 | 132'530 | 0 | **132'530** |
| Peaches and nectarines | Rosales | 1'105'919 | 341'208 | 2 | **113'736** |
| Rapeseed | Brassicales | 1'222'029 | 112'947 | 0 | **112'947** |
| Barley | Poales | 1'279'195 | 110'810 | 0 | **110'810** |
| Garlic | Asparagales | 256'365 | 107'425 | 0 | **107'425** |
| Lettuce and chicory | Asterales | 1'245'420 | 103'252 | 0 | **103'252** |
| Pumpkins, squash and gourds | Cucurbitales | 354'514 | 188'636 | 1 | **94'318** |
| Almonds, with shell | Rosales | 28'628 | 73'431 | 0 | **73'431** |
| Apricots | Rosales | 211'812 | 71'851 | 0 | **71'851** |
| Chillies and peppers, green | Solanales | 1'855'912 | 1'538'650 | 21 | **69'939** |
| Beans, green | Fabales | 495'639 | 2'190'053 | 34 | **62'573** |
| Cranberries | Ericales | 65'054 | 56'996 | 0 | **56'996** |
| Raspberries | Rosales | 159'036 | 52'208 | 0 | **52'208** |
| Rice, paddy | Poales | 56'708 | 40'891 | 0 | **40'891** |
| Linseed | Malpighiales | 291'554 | 36'457 | 0 | **36'457** |
| Plantains | Zingiberales | 109'549 | 35'747 | 0 | **35'747** |

| Commodity | Plant Order | Agr. Exports Europe, $th 03-07 | TV $th | #alien insect interceptions 03-07 | TVPI $th |
|---|---|---|---|---|---|
| Figs | Rosales | 52'835 | 34'976 | 0 | **34'976** |
| Watermelons | Cucurbitales | 369'525 | 303'296 | 8 | **33'700** |
| Coconuts | Arecales | 30'943 | 62'400 | 1 | **31'200** |
| Carrots and turnips | Apiales | 319'990 | 26'309 | 0 | **26'309** |
| Broad beans, horse beans, dry | Fabales | 46'674 | 40'682 | 1 | **20'341** |
| Palm kernels | Arecales | 8'428 | 17'837 | 0 | **17'837** |
| Cauliflowers and broccoli | Brassicales | 415'141 | 17'344 | 0 | **17'344** |
| Mangoes, mangosteens, guavas | Sapindales | 422'431 | 1'940'793 | 115 | **16'731** |
| Sweet potatoes | Solanales | 57'832 | 121'267 | 8 | **13'474** |
| Cabbages and other brassicas | Brassicales | 382'235 | 47'412 | 3 | **11'853** |
| Ginger | Zingiberales | 36'312 | 10'943 | 0 | **10'943** |
| Hazelnuts, with shell | Fagales | 27'300 | 10'191 | 0 | **10'191** |
| Artichokes | Asterales | 53'543 | 10'014 | 0 | **10'014** |
| Other melons (inc.cantaloupes) | Cucurbitales | 765'091 | 1'907'500 | 224 | **8'478** |
| String beans | Fabales | 1'178 | 7'773 | 0 | **7'773** |
| Cucumbers and gherkins | Cucurbitales | 936'879 | 33'337 | 4 | **6'667** |
| Asparagus | Asparagales | 335'910 | 40'823 | 6 | **5'832** |
| Chicory roots | Asterales | 9'006 | 3'732 | 0 | **3'732** |
| Cashew nuts, with shell | Sapindales | 11'062 | 3'656 | 0 | **3'656** |
| Oats | Poales | 111'031 | 3'556 | 0 | **3'556** |
| Blueberries | Ericales | 51'448 | 3'425 | 0 | **3'425** |
| Peas, green | Fabales | 148'299 | 742'872 | 240 | **3'082** |
| Sugar cane | Poales | 1'800 | 2'801 | 0 | **2'801** |
| Onions (inc. shallots), green | Asparagales | 40'477 | 2'733 | 0 | **2'733** |
| Anise, badian, fennel, corian. | Apiales | 66'093 | 93'594 | 40 | **2'283** |
| Kiwi fruit | Ericales | 894'044 | 1'804 | 0 | **1'804** |
| Spinach | Caryophyllales | 55'447 | 5'088 | 2 | **1'696** |
| Cinnamon (canella) | Laurales | 25'959 | 1'549 | 0 | **1'549** |
| Chestnuts | Fagales | 70'875 | 1'479 | 0 | **1'479** |
| Safflower seed | Asterales | 6'988 | 1'249 | 0 | **1'249** |
| Arecanuts | Arecales | 2'681 | 777 | 0 | **777** |
| Potatoes | Solanales | 1'673'177 | 1'410 | 1 | **705** |
| Coffee Husks and Skins | Gentianales | 2'050 | 400 | 0 | **400** |
| Quinces | Rosales | 9'748 | 399 | 0 | **399** |
| Jute | Malvales | 9'373 | 8'040 | 24 | **322** |
| Eggplants (aubergines) | Solanales | 178'330 | 49'902 | 350 | **142** |
| Olives | Lamiales | 19'354 | 136 | 0 | **136** |
| Sour cherries | Rosales | 32'042 | 133 | 0 | **133** |
| Sugar beet | Caryophyllales | 19'006 | 76 | 0 | **76** |
| Okra | Malvales | 183 | 70 | 1 | **35** |
| Gooseberries | Saxifragales | 80 | 1 | 0 | **1** |
| Taro (cocoyam) | Alismatales | 0 | 0 | 17 | **0** |
| Cassava | Malpighiales | 0 | 0 | 11 | **0** |
| Persimmons | Ericales | 0 | 0 | 1 | **0** |
| Ramie | Urticales | 0 | 0 | 0 | **0** |
| Chillies and peppers, dry | Solanales | 192'687 | 0 | 0 | **0** |
| Pistachios | Sapindales | 629'515 | 0 | 0 | **0** |
| Hops | Rosales | 84'840 | 0 | 0 | **0** |
| Hempseed | Rosales | 7'838 | 0 | 0 | **0** |
| Mixed grain | Poales | 24'510 | 0 | 0 | **0** |
| Maize for forage and silage | Poales | 0 | 0 | 0 | **0** |
| Fonio | Poales | 0 | 0 | 0 | **0** |
| Canary seed | Poales | 30'241 | 0 | 0 | **0** |
| Cloves | Myrtales | 11'815 | 0 | 0 | **0** |
| Kolanuts | Malvales | 0 | 0 | 0 | **0** |
| Kapokseed in Shell | Malvales | 0 | 0 | 0 | **0** |
| Castor oil seed | Malpighiales | 564 | 0 | 0 | **0** |

| Commodity | Plant Order | Agr. Exports Europe, $th 03-07 | TV $th | #alien insect interceptions 03-07 | TVPI $th |
|---|---|---|---|---|---|
| Nutmeg, mace and cardamoms | Magnoliales | 70'261 | 0 | 0 | **0** |
| Peppermint | Lamiales | 0 | 0 | 0 | **0** |
| Vetches | Fabales | 0 | 0 | 0 | **0** |
| Lupins | Fabales | 0 | 0 | 0 | **0** |
| Cow peas, dry | Fabales | 0 | 0 | 0 | **0** |
| Beans, dry | Fabales | 311'644 | 0 | 0 | **0** |
| Bambara beans | Fabales | 0 | 0 | 0 | **0** |
| Karite Nuts (Sheanuts) | Ericales | 0 | 0 | 0 | **0** |
| Brazil nuts, with shell | Ericales | 4'227 | 0 | 0 | **0** |
| Yams | Dioscoreales | 94 | 0 | 0 | **0** |
| Melonseed | Cucurbitales | 0 | 0 | 0 | **0** |
| Quinoa | Caryophyllales | 0 | 0 | 0 | **0** |
| Buckwheat | Caryophyllales | 10'385 | 0 | 0 | **0** |
| Mustard seed | Brassicales | 65'628 | 0 | 0 | **0** |
| Vanilla | Asparagales | 96'690 | 0 | 0 | **0** |
| Sisal | Asparagales | 19'610 | 0 | 0 | **0** |
| Agave Fibres Nes | Asparagales | 1 | 0 | 0 | **0** |
| Yautia (cocoyam) | Alismatales | 3 | 0 | 0 | **0** |
| **Agriculture Total** | | **70'900'396** | **170'157'879** | **1'167** | **145'683** |
