# Supplementary material for: Gaps in Border Controls Are Related to Quarantine Alien Insect Invasions in Europe
Source: PLoS One. 2012 Oct 24;7(10):e47689. doi: 10.1371/journal.pone.0047689 (PMC3480426; doi:10.1371/journal.pone.0047689)
Supplement: Table S6 — Control of quarantine alien insects in Europe for the 5-year period 2003 to 2007. For each quarantine alien insect, data shown are; EU1 if the species is listed in the EU Directive 2000/29/EC, EPPO2 if the spedies is listed in the EPPO quarantine lists, the Trade Volume to be inspected (TV), the number of quarantine interceptions (EPPO), and the Trade Volume to be inspected Per Interception (TVPI) (ranked). (PDF) [file pone.0047689.s007.pdf]

Table S6. Control of quarantine alien insects in Europe for the 5-year period 2003 to 2007. For each quarantine alien insect, data shown are; EU[1] if the species is listed in the EU Directive 2000/29/EC, EPPO[2] if the spedies is listed in the EPPO quarantine lists, the Trade Volume to be inspected (TV), the number of quarantine interceptions (EPPO), and the Trade Volume to be inspected Per Interception (TVPI) (ranked).

Insect Species	Family	EU[1]	EPPO[2]	TV $th	#alien insect interceptions 03-07	TVPI $th
Diabrotica barberi	Chrysomelidae	1	A1	7'619'950	0	**7'619'950**
Aleurocanthus woglumi	Aleyrodidae	0	A1	6'850'746	0	**6'850'746**
Maconellicoccus hirsutus	Pseudococcidae	0	A1	5'706'971	0	**5'706'971**
Anastrepha fraterculus	Tephritidae	1	A1	5'689'580	0	**5'689'580**
Liriomyza trifolii	Agromyzidae	0	A2	5'677'971	0	**5'677'971**
Metamasius hemipterus	Curculionidae	0	A2	4'616'336	0	**4'616'336**
Helicoverpa zea	Noctuidae	1	A1	8'129'918	1	**4'064'959**
Rhynchophorus palmarum	Curculionidae	0	A1	3'726'549	0	**3'726'549**
Opogona sacchari	Tineidae	0	A2	3'535'994	0	**3'535'994**
Unaspis citri	Diaspididae	0	A1	3'221'669	0	**3'221'669**
Frankliniella occidentalis	Thripidae	0	A2	3'129'205	0	**3'129'205**
Spodoptera eridania	Noctuidae	1	A1	2'509'369	0	**2'509'369**
Spodoptera frugiperda	Noctuidae	1	A1	7'294'841	2	**2'431'614**
Tetranychus evansi	Acari	0	A2	2'327'549	0	**2'327'549**
Liriomyza sativae	Agromyzidae	1	A2	6'906'826	2	**2'302'275**
Pheletes californicus	Elateridae	0	A1	2'220'198	0	**2'220'198**
Cacoecimorpha pronubana	Tortricidae	0	A2	2'137'220	0	**2'137'220**
Anastrepha ludens	Tephritidae	1	A1	1'953'720	0	**1'953'720**
Carneocephala fulgida	Cicadellidae	1	A1	1'910'399	0	**1'910'399**
Diabrotica virgifera	Chrysomelidae	1	A2	1'879'737	0	**1'879'737**
Epitrix similaris	Chrysomelidae	0	A2	1'789'404	0	**1'789'404**
Quadraspidiotus perniciosus	Diaspididae	0	A2	1'537'646	0	**1'537'646**
Ceratitis capitata	Tephritidae	0	A2	9'119'303	5	**1'519'884**
Melanotus communis	Elateridae	0	A1	1'457'356	0	**1'457'356**
Diabrotica undecimpunctata	Chrysomelidae	1	A1	1'329'554	0	**1'329'554**
Halyomorpha halys	Pentatomidae	0	Alert	1'320'174	0	**1'320'174**
Aleurocanthus spiniferus	Aleyrodidae	0	A2	1'268'916	0	**1'268'916**
Popillia japonica	Scarabaeidae	0	A2	1'205'291	0	**1'205'291**
Naupactus leucoloma	Curculionidae	1	A1	1'183'302	0	**1'183'302**
Pterandrus rosa	Tephritidae	1	-	1'172'808	0	**1'172'808**
Myndus crudus	Cixiidae	1	A1	1'148'477	0	**1'148'477**
Homalodisca coagulata	Cicadellidae	0	A1	1'068'154	0	**1'068'154**
Heteronychus arator	Scarabaeidae	0	A1	1'003'902	0	**1'003'902**
Listronotus bonariensis	Curculionidae	0	A1	998'412	0	**998'412**
Rhagoletis pomonella	Tephritidae	1	A1	971'405	0	**971'405**
Epitrix cucumeris	Chrysomelidae	0	A1	953'717	0	**953'717**
Anthonomus grandis	Curculionidae	0	A1	933'135	0	**933'135**
Scirtothrips aurantii	Thripidae	0	A1	837'561	0	**837'561**
Toxoptera citricida	Aphididae	0	A2	835'846	0	**835'846**
Eutetranychus orientalis	Acari	0	A2	825'316	0	**825'316**
Tuta absoluta	Gelechiidae	0	A2	802'503	0	**802'503**
Epitrix tuberis	Chrysomelidae	0	A1	787'382	0	**787'382**
Anthonomus eugenii	Curculionidae	0	A1	753'683	0	**753'683**
Spodoptera littoralis	Noctuidae	0	A2	5'175'787	6	**739'398**
Trogoderma granarium	Dermestidae	0	A2	725'925	0	**725'925**
Ceratitis rosa	Tephritidae	0	A1	1'998'243	2	**666'081**
Bactrocera invadens	Tephritidae	0	A1	634'114	0	**634'114**
Blitopertha orientalis	Scarabaeidae	0	A1	583'983	0	**583'983**
Viteus vitifoliae	Phylloxeridae	0	A2	580'340	0	**580'340**

Insect Species	Family	EU[1]	EPPO[2]	TV $th	#alien insect interceptions 03-07	TVPI $th
Pardalaspis quinaria	Tephritidae	1	-	400'862	0	**400'862**
Trioza erytreae	Triozidae	0	A1	399'453	0	**399'453**
Bactrocera tryoni	Tephritidae	0	A1	346'369	0	**346'369**
Bactrocera papayae	Tephritidae	0	A1	305'344	0	**305'344**
Spodoptera litura	Noctuidae	1	A1	2'331'671	9	**233'167**
Liriomyza huidobrensis	Agromyzidae	0	A2	4'447'251	19	**222'363**
Anastrepha obliqua	Tephritidae	1	A1	4'434'567	20	**211'170**
Bactrocera carambolae	Tephritidae	0	A1	203'772	0	**203'772**
Draeculacephala minerva	Cicadellidae	1	A1	186'143	0	**186'143**
Graphocephala atropunctata	Cicadellidae	1	A1	177'222	0	**177'222**
Anastrepha suspensa	Tephritidae	1	A1	170'942	0	**170'942**
Diocalandra frumenti	Curculionidae	0	Alert	165'100	0	**165'100**
Dryocosmus kuriphilus	Cynipidae	0	A2	156'914	0	**156'914**
Rhagoletis suavis	Tephritidae	1	-	156'425	0	**156'425**
Rhagoletis completa	Tephritidae	1	-	156'425	0	**156'425**
Diaphorina citri	Psyllidae	0	A1	147'955	0	**147'955**
Scirtothrips citri	Thripidae	0	A1	143'000	0	**143'000**
Nemorimyza maculosa	Agromyzidae	0	A1	135'507	0	**135'507**
Amauromyza maculosa	Agromyzidae	1	-	135'127	0	**135'127**
Conotrachelus nenuphar	Curculionidae	1	A1	120'864	0	**120'864**
Scirtothrips dorsalis	Thripidae	0	A2	1'201'239	9	**120'124**
Bemisia tabaci	Aleyrodidae	1	A2	12'666'466	120	**104'682**
Anomala orientalis	Scarabaeidae	1	-	100'763	0	**100'763**
Cydia packardi	Tortricidae	0	A1	89'217	0	**89'217**
Cydia prunivora	Tortricidae	0	A1	80'257	0	**80'257**
Malacosoma americanum	Lasiocampidae	0	A1	73'912	0	**73'912**
Rhizoecus hibisci	Pseudococcidae	0	A1	73'486	0	**73'486**
Choristoneura rosaceana	Tortricidae	1	A1	62'843	0	**62'843**
Sternochetus mangiferae	Curculionidae	0	A1	52'157	0	**52'157**
Bactrocera cucurbitae	Tephritidae	0	A1	272'497	6	**38'928**
Rhynchophorus ferrugineus	Curculionidae	0	A2	66'510	1	**33'255**
Bactrocera dorsalis	Tephritidae	0	A1	1'094'568	35	**30'405**
Bactrocera dorsalis	Tephritidae	0	A1	1'094'568	35	**30'405**
Diaphania indica	Crambidae	0	A2	1'612'798	59	**26'880**
Anthonomus signatus	Curculionidae	0	A1	24'990	0	**24'990**
Rhagoletis cingulata	Tephritidae	1	A2	22'005	0	**22'005**
Rhagoletis indifferens	Tephritidae	1	A1	21'976	0	**21'976**
Rhagoletis fausta	Tephritidae	1	A1	21'976	0	**21'976**
Leptinotarsa decemlineata	Chrysomelidae	0	A2	16'273	0	**16'273**
Thrips palmi	Thripidae	1	A1	4'318'734	373	**11'547**
Helicoverpa armigera	Noctuidae	0	A2	3'113'574	275	**11'281**
Bactrocera pyrifoliae	Tephritidae	0	A1	9'543	0	**9'543**
Rhagoletis mendax	Tephritidae	1	A1	8'474	0	**8'474**
Bactrocera caryae	Tephritidae	0	A1	5'000	0	**5'000**
Dacus ciliatus	Tephritidae	1	A1	26'146	8	**2'905**
Bactrocera zonata	Tephritidae	0	A1	102'626	49	**2'053**
Bactrocera occipitalis	Tephritidae	0	A1	1'920	0	**1'920**
Bactrocera philippinensis	Tephritidae	0	A1	1'767	0	**1'767**
Bactrocera kandiensis	Tephritidae	0	A1	1'341	0	**1'341**
Erschoviella musculana	Noctuidae	0	A2	902	0	**902**
Carposina sasakii	Carposinidae	0	A2	594	0	**594**
Leucinodes orbonalis	Pyralidae	0	Alert	79'912	135	**588**
Paysandisia archon	Castniidae	0	A2	454	0	**454**
Numonia pyrivorella	Geometridae	0	A2	414	0	**414**
Lymantria mathura	Lymantriidae	0	A2	241	0	**241**
Pardalaspis cyanescens	Tephritidae	1	-	218	0	**218**
Anthonomus bisignifer	Curculionidae	0	A1	123	0	**123**

Insect Species	Family	EU[1]	EPPO[2]	TV $th	#alien insect interceptions 03-07	TVPI $th
Bactrocera tsuneonis	Tephritidae	0	A1	89	0	**89**
Tecia solanivora	Gelechiidae	0	A2	38	0	**38**
Cydia inopinata	Tortricidae	0	A2	34	0	**34**
Premnotrypes vorax	Curculionidae	1	A1	16	0	**16**
Premnotrypes suturicallus	Curculionidae	1	A1	16	0	**16**
Premnotrypes latithorax	Curculionidae	1	A1	16	0	**16**
Bactrocera minax	Tephritidae	0	A1	13	0	**13**
Bactrocera cucumis	Tephritidae	0	A1	10	0	**10**
Rhagoletis ribicola	Tephritidae	1	-	0	0	**0**
Epochra canadensis	Tephritidae	1	-	0	0	**0**
Cryptophlebia leucotreta	Tortricidae	0	A2	0	32	**0**
Xylosandrus crassiusculus	Scolytidae	0	Alert	0	0	**0**
Ips subelongatus	Scolytidae	0	A2	0	0	**0**
Monochamus galloprovincialis	Cerambycidae	1	-	0	0	**0**
Tetropium gracilicorne	Cerambycidae	0	A2	0	0	**0**
Monochamus urussovii	Cerambycidae	1	-	0	0	**0**
Monochamus saltuarius	Cerambycidae	1	A1	0	0	**0**
Xylotrechus altaicus	Cerambycidae	0	A2	0	0	**0**
Scolytus morawitzi	Scolytidae	0	A2	0	0	**0**
Ips hauseri	Scolytidae	0	A2	0	0	**0**
Sirex ermak	Siricidae	0	A2	0	0	**0**
Monochamus sutor	Cerambycidae	1	A1	0	0	**0**
Agrilus planipennis	Buprestidae	0	A2	0	0	**0**
Anoplophora chinensis	Cerambycidae	1	A2	0	0	**0**
Arrhenodes minutus	Curculionidae	1	A1	0	0	**0**
Saperda candida	Cerambycidae	0	A1	0	0	**0**
Enaphalodes rufulus	Cerambycidae	0	Alert	0	0	**0**
Agrilus anxius	Buprestidae	0	Alert	0	0	**0**
Pseudopityophthorus minutissimus	Scolytidae	1	A1	0	0	**0**
Anoplophora glabripennis	Cerambycidae	1	A1	0	0	**0**
Lopholeucaspis japonica	Diaspididae	0	A2	0	0	**0**
Dendrolimus sibiricus	Lasiocampidae	0	A2	0	0	**0**
Hesperophanes campestris	Cerambycidae	0	A2	0	0	**0**
Megaplatypus mutatus	Platypodidae	0	A2	0	0	**0**
Malacosoma paralella	Lasiocampidae	0	A2	0	0	**0**
Lepidosaphes ussuriensis	Diaspididae	0	A2	0	0	**0**
Ips calligraphus	Scolytidae	0	A1	0	0	**0**
Monochamus scutellatus	Cerambycidae	1	A1	0	0	**0**
Monochamus carolinensis	Cerambycidae	1	A1	0	0	**0**
Ips pini	Scolytidae	0	A1	0	0	**0**
Gnathotrichus sulcatus	Scolytidae	0	A1	0	0	**0**
Dendroctonus rufipennis	Scolytidae	0	A1	0	0	**0**
Dendroctonus pseudotsugae	Scolytidae	0	A1	0	0	**0**
Dendroctonus ponderosae	Scolytidae	0	A1	0	0	**0**
Dendroctonus brevicomis	Scolytidae	0	A1	0	0	**0**
Ips grandicollis	Scolytidae	0	A1	0	0	**0**
Monochamus titillator	Cerambycidae	1	A1	0	0	**0**
Monochamus obtusus	Cerambycidae	1	A1	0	0	**0**
Monochamus notatus	Cerambycidae	1	A1	0	0	**0**
Monochamus mutator	Cerambycidae	1	A1	0	0	**0**
Monochamus marmorator	Cerambycidae	1	A1	0	0	**0**
Monochamus clamator	Cerambycidae	1	-	0	0	**0**
Ips plastographus	Scolytidae	0	A1	0	0	**0**
Dryocoetes confusus	Scolytidae	0	A1	0	0	**0**
Malacosoma disstria	Lasiocampidae	0	A1	0	0	**0**
Scaphoideus luteolus	Cicadellidae	1	A1	0	0	**0**
Choristoneura conflictana	Tortricidae	1	A1	0	0	**0**

Insect Species	Family	EU[1]	EPPO[2]	TV $th	#alien insect interceptions 03-07	TVPI $th
Ips lecontei	Scolytidae	0	A1	0	0	**0**
Dendroctonus frontalis	Scolytidae	0	A1	0	0	**0**
Monochamus rubigeneus	Cerambycidae	1	-	0	0	**0**
Dendroctonus adjunctus	Scolytidae	0	A1	0	0	**0**
Ips confusus	Scolytidae	0	A1	0	0	**0**
Ips paraconfusus	Scolytidae	0	A1	0	0	**0**
Pissodes nemorensis	Curculionidae	0	A1	0	0	**0**
Pissodes terminalis	Curculionidae	0	A1	0	0	**0**
Pissodes strobi	Curculionidae	0	A1	0	0	**0**
Orgyia pseudotsugata	Lymantriidae	0	A1	0	0	**0**
Choristoneura occidentalis	Tortricidae	1	A1	0	0	**0**
Choristoneura fumiferana	Tortricidae	1	A1	0	0	**0**
Oligonychus perditus	Acari	0	A1	0	0	**0**
Aeolesthes sarta	Cerambycidae	0	A2	0	0	**0**
Anoplophora malasiaca	Cerambycidae	1	-	0	0	**0**
Psacothea hilaris	Cerambycidae	0	Alert	0	0	**0**
Xylotrechus namanganensis	Cerambycidae	0	A2	0	0	**0**
Monochamus alternatus	Cerambycidae	1	A1	0	0	**0**
Chrysophtharta bimaculata	Chrysomelidae	0	Alert	0	0	**0**
Monochamus tesserula	Cerambycidae	1	-	0	0	**0**
Monochamus nitens	Cerambycidae	1	A1	0	0	**0**
Drosophila suzukii	Drosophilidae	0	Alert	0	0	**0**
Diaphania perspectalis	Pyralidae	0	Alert	0	0	**0**
Strobilomyia viaria	Anthomyidae	0	A2	0	0	**0**
Rhacochlaena japonica	Tephritidae	1	-	0	0	**0**
Monochamus sartor	Cerambycidae	1	-	0	0	**0**
Margarodes vredendalensis	Margarodidae	0	A1	0	0	**0**
Margarodes vitis	Margarodidae	0	A1	0	0	**0**
Margarodes prieskaensis	Margarodidae	0	A1	0	0	**0**
Gonipterus scutellatus	Curculionidae	0	A2	0	0	**0**
Gonipterus gibberus	Curculionidae	0	A1	0	0	**0**
Diabrotica speciosa	Chrysomelidae	0	A1	0	0	**0**
Dendrolimus superans	Lasiocampidae	0	A2	0	0	**0**
Cacyreus marshalli	Lycaenidae	0	A2	0	0	**0**
Aculops fuchsiae	Acari	0	A2	0	0	**0**
Acleris variana	Tortricidae	1	A1	0	0	**0**
Acleris gloverana	Tortricidae	1	A1	0	0	**0**
**World Total**				**170'157'879**	**1203**	**141'327**
