# Supplementary material for: Gaps in Border Controls Are Related to Quarantine Alien Insect Invasions in Europe
Source: PLoS One. 2012 Oct 24;7(10):e47689. doi: 10.1371/journal.pone.0047689 (PMC3480426; doi:10.1371/journal.pone.0047689)
Supplement: Table S7 — European country insect invasions data for the 5-year period 2003 to 2007. For each European country, data shown are; the total value of agricultural imports (US$th, FAO), the Trade Volume to be inspected (TV), the number of quarantine alien insect interceptions (EPPO), the Trade Volume to be inspected Per Interception (TVPI) (ranked), the number of quarantine listed alien insects that have established (DAISIE, EPPO), the Nominal GDP ($, IMF), Agriculture as a % of GDP, Capital city latitude (degrees), Country area (km2)and altitudinal range (m). (PDF) [file pone.0047689.s008.pdf]

Table S7. European country insect invasions data for the 5-year period 2003 to 2007. For each European country, data shown are; the total value of agricultural imports (US$th, FAO), the Trade Volume to be inspected (TV), the number of quarantine alien insect interceptions (EPPO), the Trade Volume to be inspected Per Interception (TVPI) (ranked), the number of quarantine listed alien insects that have established (DAISIE, EPPO), the Nominal GDP ($, IMF), Agriculture as a % of GDP, Capital city latitude (degrees), Country area (km^2)and altitudinal range (m)

Country	Agricultural imports, $th	TV $th	#aliens int. 03-07	TVPI $th	#alien insects Establ.	Nominal GDP, $ 03-07	Agr. as % GDP	(Deg.) Capital Latitude	(km^2) Country Area	(m) Altitiude Range
Portugal	1'729'004	5'609'972	0	**5'609'972**	25	18'450'666	2.9%	38.5	92'090	2'351
Switzerland	1'619'704	3'337'449	0	**3'337'449**	20	50'650'884	1.5%	46.6	41'285	4'439
Italy	5'953'086	15'373'298	4	**3'074'660**	33	31'000'044	2.1%	41.5	301'338	4'813
Greece	1'158'012	2'930'451	0	**2'930'451**	15	22'475'547	3.4%	38.0	131'990	2'919
Belgium	6'529'745	16'795'978	5	**2'799'330**	7	36'273'866	0.8%	50.5	30'528	694
Poland	1'577'412	1'852'699	0	**1'852'699**	12	8'076'329	4.6%	52.1	312'685	2'501
Romania	622'093	1'536'906	0	**1'536'906**	13	4'885'133	12.4%	44.3	238'391	2'544
Finland	647'598	932'001	0	**932'001**	5	38'159'573	3.4%	60.1	338'424	1'324
Austria	1'506'736	769'602	0	**769'602**	10	37'472'092	1.7%	48.1	83'855	3'683
Spain	5'642'107	21'398'899	33	**629'379**	26	26'592'362	3.4%	40.3	504'030	3'718
Germany	14'227'833	25'101'581	54	**456'392**	16	34'613'903	0.9%	52.3	357'021	2'966
Bulgaria	195'101	455'705	0	**455'705**	14	3'736'748	7.5%	42.4	110'994	2'925
Slovenia	287'107	444'514	0	**444'514**	14	18'463'539	2.3%	46.0	20'273	2'864
Hungary	399'209	426'160	0	**426'160**	14	10'855'061	3.4%	47.3	93'030	938
Lithuania	284'954	421'317	0	**421'317**	6	8'000'747	5.3%	54.4	65'200	295
Sweden	1'427'967	1'832'266	5	**305'378**	4	42'163'735	1.6%	58.0	449'964	2'106
Cyprus	141'527	279'652	0	**279'652**	10	23'013'406	2.1%	35.1	9'248	1'951
Czech Rep.	850'671	799'404	2	**266'468**	13	12'533'234	2.8%	50.1	78'866	1'487
Denmark	1'183'889	1'735'284	8	**192'809**	8	48'009'845	4.6%	55.4	43'075	178
Malta	62'099	163'902	0	**163'902**	12	15'141'846	1.7%	35.5	316	253
Slovakia	347'003	127'350	0	**127'350**	9	9'447'673	7.8%	48.1	49'035	2'561
France	6'327'177	11'947'523	94	**125'763**	24	35'550'725	2.1%	48.5	674'843	4'812
Estonia	119'997	106'593	0	**106'593**	5	11'028'602	3.0%	59.3	45'228	318
Latvia	162'049	65'859	0	**65'859**	5	7'799'890	3.6%	56.6	64'589	312
UK	8'120'193	22'136'632	366	**60'318**	10	38'502'636	1.2%	51.3	243'610	1'348
Netherlands	8'866'520	32'937'855	602	**54'623**	10	39'854'964	1.9%	52.2	41'848	884
Luxembourg	233'383	39'624	0	**39'624**	6	83'596'662	0.4%	49.4	2'586	427
Ireland	678'221	599'405	30	**19'336**	3	49'387'888	5.0%	53.2	84'421	1'038
**Europe**	**70'900'396**	**170'157'879**	**1'203**	**141'327**						
